# Supplementary material for: Hopanoid lipids promote soybean–Bradyrhizobium symbiosis
Source: mBio. 2024 Mar 6;15(4):e02478-23. doi: 10.1128/mbio.02478-23 (PMC11005386; doi:10.1128/mbio.02478-23)
Supplement: Supplemental Figures — Supplemental table captions and Figures S1 to S11. [file mbio.02478-23-s0001.docx]

**Supplementary Material**

Pan H, Shim A, Lubin MB, Belin BJ (2023). Hopanoid lipids promote soybean-*Bradyrhizobium* symbiosis

# Supplementary Tables (see Excel files)

**Table S1. Primer sequences used for qRT-PCR.**

Primers are designed to target conserved regions of each gene that are 100-200 bp in length. “RT1” and “RT2” represent forward and reverse primers, respectively.

**Table S2. Complete RNA-seq read counts of all treatments/replicates.**

Normalized read counts of WT and Pcu-*shc* with and without cumate from all replicates of RNA-seq. Raw reads were normalized by the DESeq2’s size factor and batch factor to correct for variability of the sequencing depth and batch effect from independent experiments.

**Table S3. Relative expression of Pcu-*shc*-C compared to WT-C.**

The ratio of normalized read counts between Pcu-*shc*-C and WT-C was calculated as foldchange (fc). The log_2_fc, p-value and adjusted p-value (false discovery rate; FDR) of the filtered genes (8155) with all counts more than 12 in all samples are listed. Non-differentially expressed genes (NoDiff) are those genes with -1<log2fc<1 and FDR≥0.05.

**Table S4. Relative expression of Pcu-*shc*+C compared to WT+C.**

Relative gene expression of Pcu-*shc*+C compared to WT+C. Parameters are as described for Table S3.

**Table S5. Up-regulated DEGs in the Venn diagram.**

Among the genes in Table S3-4, up-regulated differential expressed genes (log2fc ≥1 and FDR≤0.05) that were used to construct the Venn diagram in Fig. 5B.

**Table S6. Down-regulated DEGs in the Venn diagram**

Among the genes in Table S3-4, down-regulated differential expressed genes (log2fc ≥1 and FDR≤0.05) that were used to construct the Venn diagram in Fig. 5B.

**Table S7. Relative expression of WT+C compared to WT-C.**

Relative gene expression of WT+C compared to WT-C. Parameters are as described for Table S3.

**Table S8. Relative expression of Pcu-*shc*+C compared to Pcu-*shc*-C.**

Relative gene expression of Pcu-*shc*+C compared to Pcu-*shc*-C. Parameters are as described for Table S3.

**Table S9. qRT-PCR information for verification of RNA-seq data.**

Relative expression of representative genes (with functions of nitrogen metabolism, flagellar motility, type III secretion systems, etc.) quantified by qRT-PCR (ΔΔCt method with an internal control of 16S rRNA). The log_2_fc (mean ± standard error) of qRT-PCR are lists. Genes in red, blue and gray are up-regulated, down-regulated and non-significant DEGs, respectively, in Pcu-*shc-C* compared to WT-C.

# Supplementary Figures

#
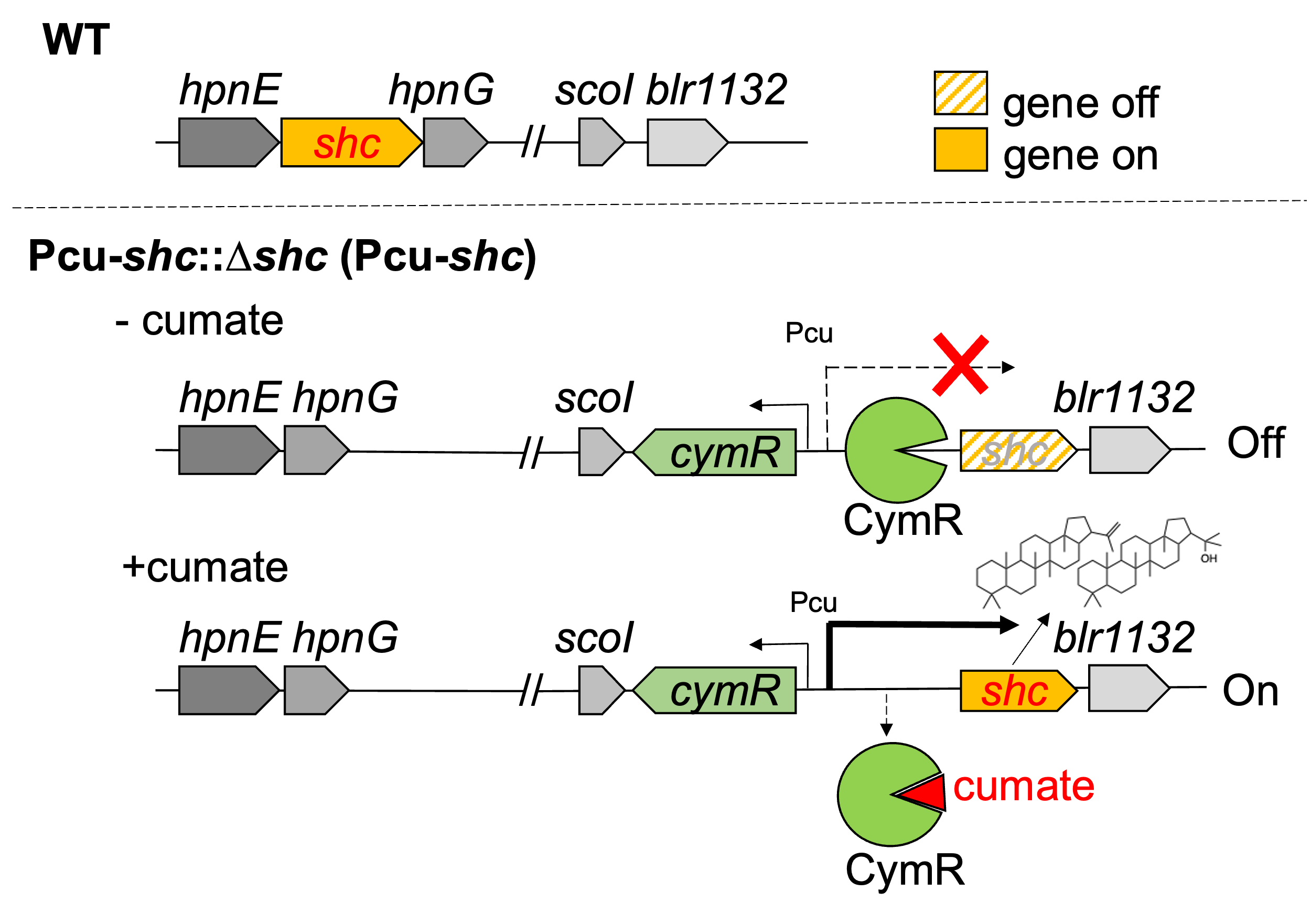


# Figure S1. Schematic of WT *B. diazoefficiens* and the cumate-inducible Pcu-*shc* strain. The exogenous cumate-inducible system was integrated into the genomic region between *scoI* (*blr1131*) and *blr1132* in a background of *shc* deletion. This system includes a copy of *shc* driven by a cumate-inducible promoter (Pcu) and a transcriptional repressor gene *cymR* driven by a constitutive promoter.

**Figure S2. Growth curves (OD_600_) of WT and Pcu-*shc* under varying cumate concentrations.** *B.diazoefficiens* WT and Pcu-*shc* with and without cumate were grown for 4 days in AG media at pH of 6.6, 30˚C supplemented with EtOH only or with (**A**) 5 µM, (**B**)10 µM, (**C**) 50 µM, (**D**) 100 µM cumate

**Figure S3. Pcu-*shc* is an inefficient soybean symbiont at 24 dpi.**

**(A)** Comparison of growth of soybean inoculated with *B.diazoefficiens* WT and Pcu-*shc* with and without cumate. Non-inoculated (NI) soybeans are shown as controls. N=9 plants/treatment. **(B)** Median and quartiles of shoot height (cm) per plant in (A). **(C)** Images of nodules in 1.5 mL Eppendorf tubes collected from each plant in (A). (**D-E**) Median and quartiles of nodule number (C) and nodule dry weight (mg) per plant (D) for the treatments in (A). **(F-H)** GC-MS quantification of nitrogen fixation rate per plant by Acetylene Reduction Assay (ARA) normalized by reaction time (F), nodule number (G), and nodule weight (H). **(I-L)** ImageJ quantification of the portion of the infection zone (IZ) containing “LIVE” (SYTO 9) and “DEAD” (PI) bacteroids (I), and the intensity of “LIVE” (J), “DEAD” (K), and combined bacterial cells (L), a.u. = arbitrary units. Statistical analysis is described in Fig 3 and the Methods section.

**Figure S4. Pcu-*shc* is an inefficient soybean symbiont at 45 dpi.**

**(A)** Comparison of growth of soybeans inoculated with *B.diazoefficiens* WT and Pcu-*shc* with and without cumate at 45 dpi. n=9 plants/treatment. **(B)** Median and quartiles of shoot height (cm) per plant in (A). **(C)** Median and quartiles of nitrogen fixation rate per plant quantified by GC-MS. **(D)** Images of nodules in 2 ml Eppendorf tubes collected from each plant in (A). (**E-F**) Median and quartiles of nodule number per plant and nodule dry weight (mg) per plant for the treatments of (A). Statistical analysis is described in Fig. 3 and the Methods section

**
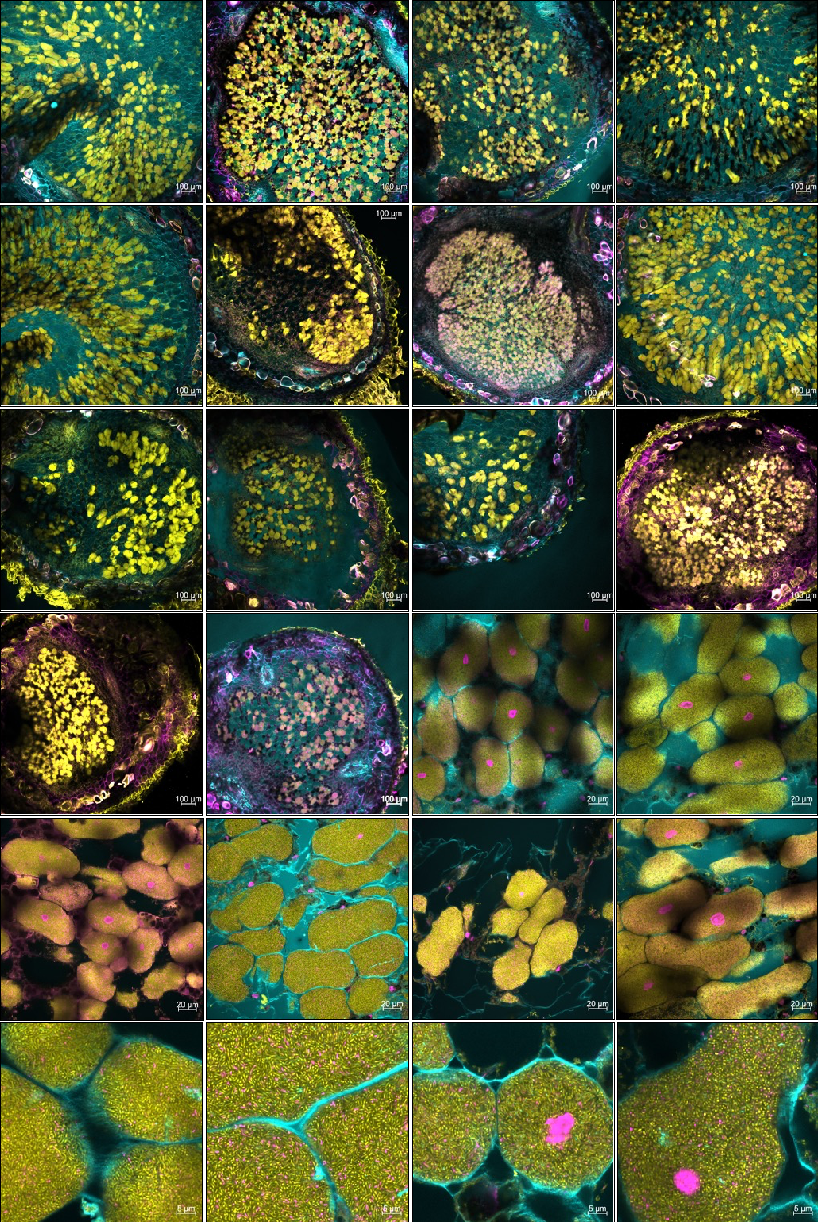
**

**Figure S5. Confocal sections of WT-C-infected soybean nodules at 27dpi.**

Nodule sections were stained with Calcofluor (cyan), SYTO 9 (yellow), and propidium iodide (magenta). Nodules were harvested from 5 plants.

**
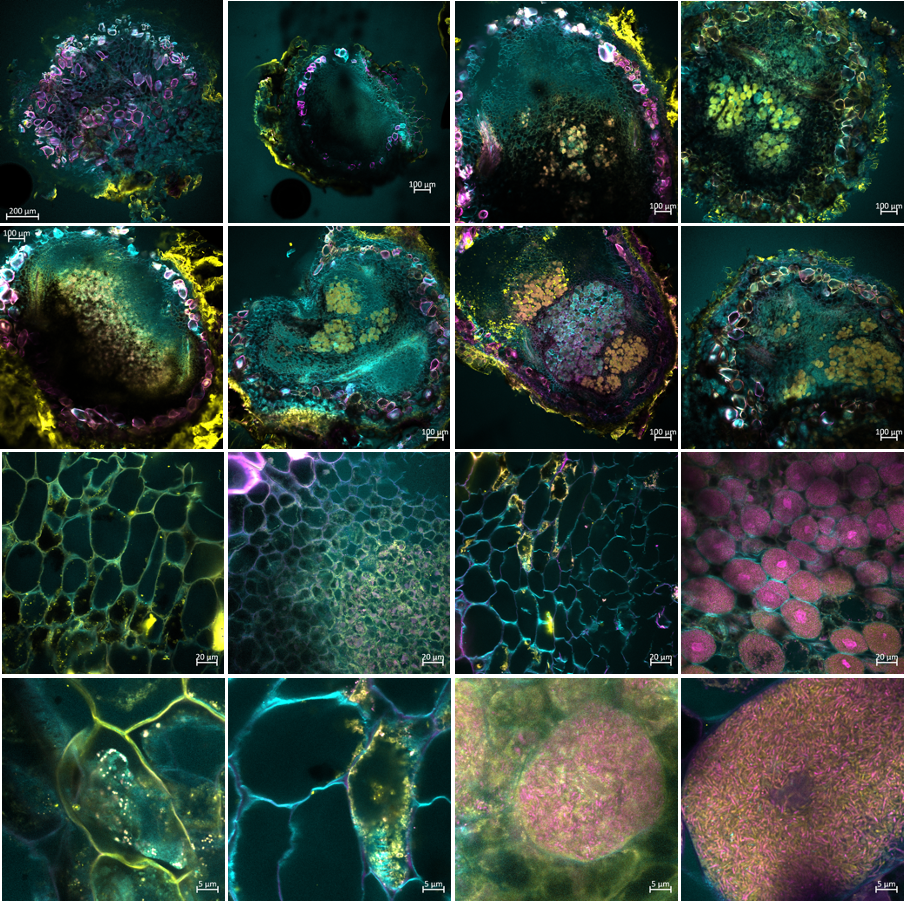
**

**Figure S6. Confocal sections of low-occupancy soybean nodules infected with Pcu-*shc*-C at 27dpi.** Nodule sections were stained with Calcofluor (cyan), SYTO 9 (yellow), and propidium iodide (magenta). Nodules were harvested from 5 plants.

**
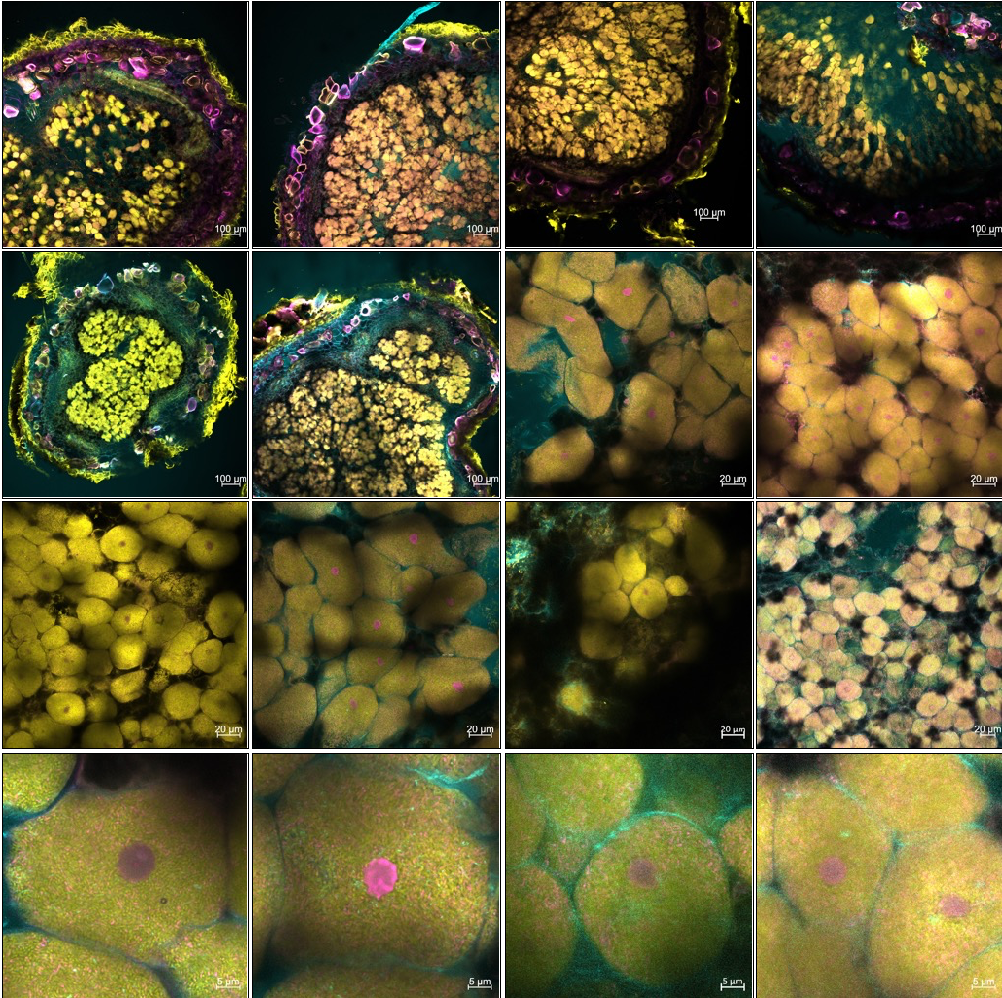
**

**Figure S7. Confocal sections of high-occupancy soybean nodules infected with Pcu-*shc*-C at 27dpi.** Nodule sections were stained with Calcofluor (cyan), SYTO 9 (yellow), and propidium iodide (magenta). Nodules were harvested from 5 plants.

**Figure S8. Pcu-*shc*-C fails to form symbioses with *Aeschynomene afraspera* at 28 dpi.**

**(A-C)** Shoot height, nodule number, and nodule dry mass at 28 dpi for *A.afraspera* inoculated with *B.diazoefficiens* WT-C or Pcu-*shc*-C (9 plants/strain). **(D-F)** Images of whole plants and roots from inoculated WT-C or Pcu-shc-C plants. **(G)** Confocal images of nodule cross-sections from plants inoculated with Pcu-*shc*-C.

**
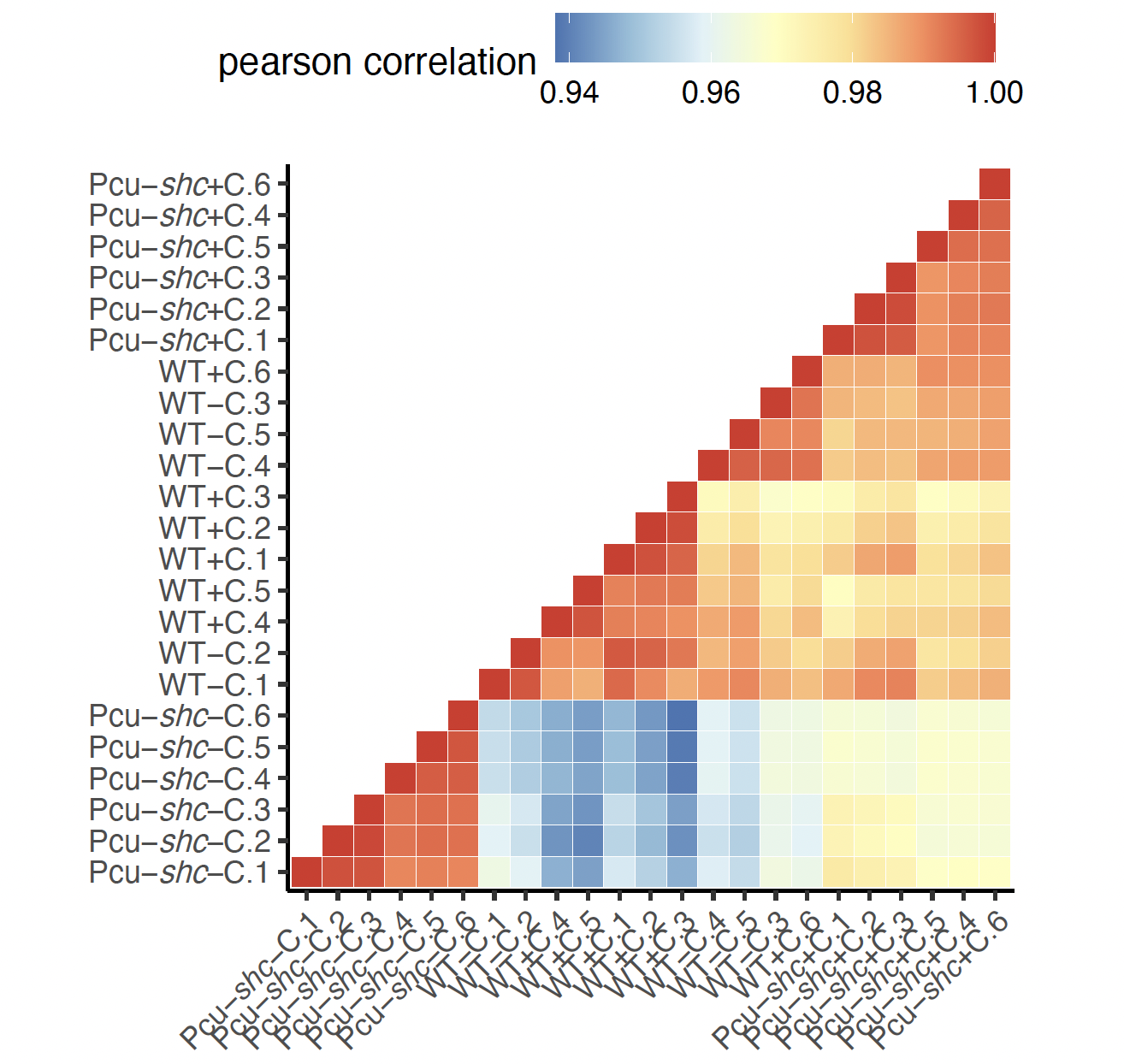
**

**Figure S9. Pearson correlation of WT and Pcu-*shc* with and without cumate.**

Pierson correlations of all RNA-seq replicates and treatments were analyzed by Deseq2 in Rstudio and were visualized by ImagGP (https://www.bic.ac.cn/ImageGP/)

**Figure S10. Volcano plots of DEGs due to cumate treatment of WT (A) and cumate-induced *shc* expression in Pcu-*shc* (B).** All genes based on read fold change (fc) and false discovery rate (FDR) of WT+C compared to WT-C **(A)** and Pcu-*shc*+C compared to Pcu-*shc*-C **(B)**. The red dots represent up-regulated genes (log2fc≥1, FDR <0.05), the blue dots represent down-regulated genes (log2fc≤-1, FDR<0.05), and the gray dots represent non-differentially expressed genes (-1<log2fc<1, FDR≥0.05).

**
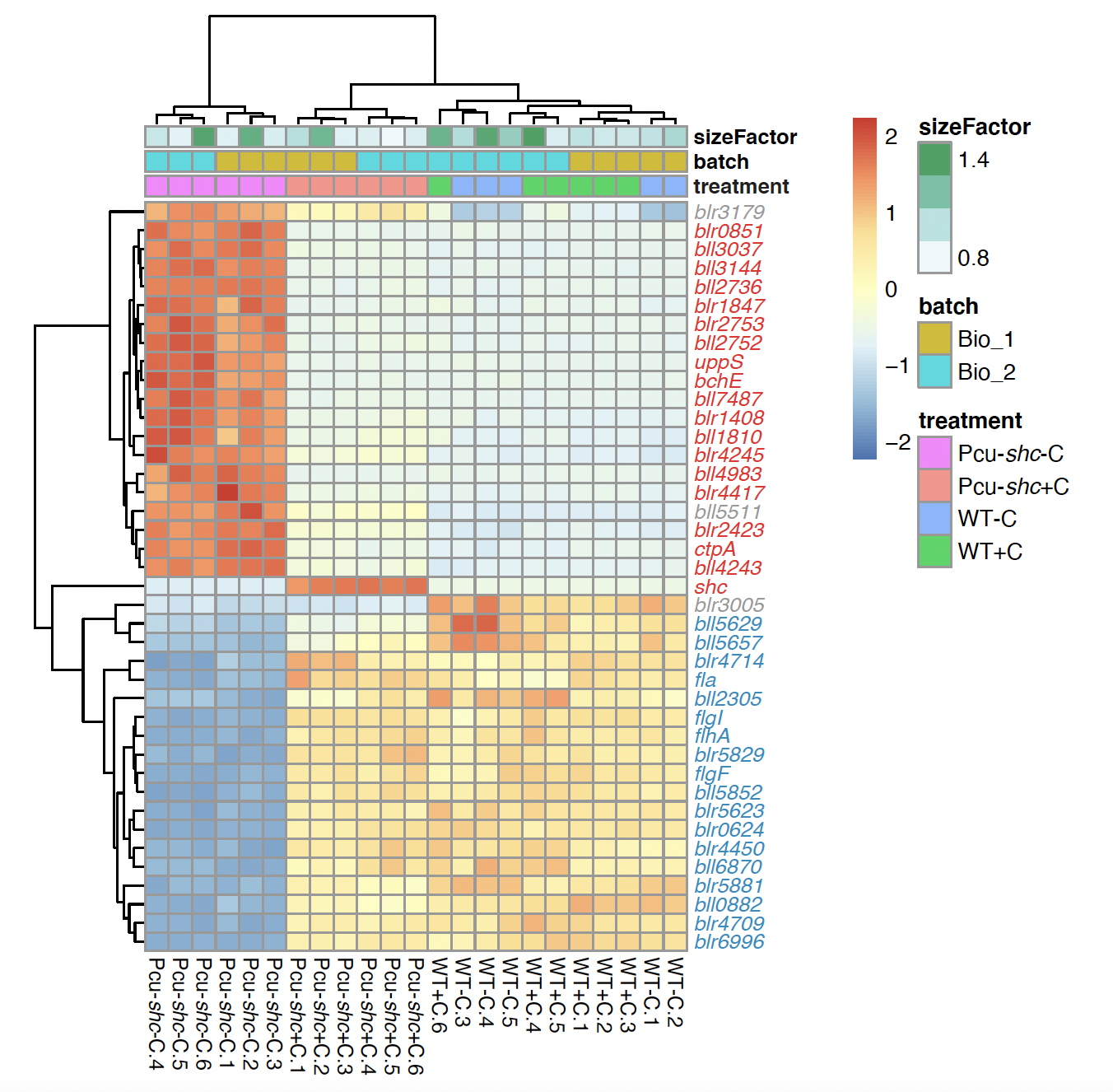
**

**Figure S11. Top 20 up and down-regulated DEGs in Pcu-*shc*-C compared to WT-C**.

Raw counts of WT and Pcu-*shc* with and without cumate from RNA-seq were normalized by the DESeq2’s size factor and batch factor to correct for variability of the sequencing depth and batch effect from independent experiments. Heatmap was made by ImagGP from the top 20 up-and down-regulated DEGs of Pcu-*shc*-C compared to WT-C as shown in Table S4-S5, ranking by FDR. Bio_1 and Bio_2 represent two batches of samples collected from 09/2022 and 10/2022 respectively. Up-regulated genes are marked in red and down-regulate genes in blue. The false positive genes due to cumate or construct effects are marked in grey.
